# Intrinsic Capacity vs. Multimorbidity: A Function-Centered Construct Predicts Disability Better Than a Disease-Based Approach in a Community-Dwelling Older Population Cohort

**DOI:** 10.3389/fmed.2021.753295

**Published:** 2021-09-28

**Authors:** Jing Zhao, Jagadish K. Chhetri, Yi Chang, Zheng Zheng, Lina Ma, Piu Chan

**Affiliations:** ^1^Department of Geriatrics, Neurology and Neurobiology, National Clinical Research Center for Geriatric Disease, Xuanwu Hospital of Capital Medical University, Beijing, China; ^2^Department of Respiration, Xuanwu Hospital of Capital Medical University, Beijing, China; ^3^Key Laboratory for Neurodegenerative Disease of the Ministry of Education, Beijing Key Laboratory for Parkinson's Disease, Parkinson Disease Center of Beijing Institute for Brain Disorders, Beijing, China; ^4^Clinical Center for Parkinson's Disease, Capital Medical University, Beijing, China; ^5^Advanced Innovation Center for Human Brain Protection, Capital Medical University, Beijing, China

**Keywords:** ICOPE, integrated care, comorbidity, aging, older Chinese

## Abstract

**Objective:** This study aimed to assess the status of intrinsic capacity (IC)—a novel function-centered construct proposed by the WHO and examine whether impairment in IC predicts subsequent 1-year activities of daily living (ADL) disability better than a disease-based approach, i. e., multimorbidity status.

**Methods:** This study included data of community-dwelling older adults from the Beijing Longitudinal Study on Aging II aged 65 years or older who were followed up at 1 year. Multivariate logistic regressions were performed to estimate the odds of ADL disability at baseline and 1-year follow-up.

**Results:** A total of 7,298 older participants aged 65 years or older were included in the current study. About 4,742 older adults were followed up at 1 year. At baseline, subjects with a higher impairment in IC domains showed higher odds of ADL disability [adj. odds ratio (OR) = 9.51 for impairment in ≥3 domains, area under the curve (AUC) = 0.751] compared to much lower odds of ADL disability in subjects with a higher number (≥3) of chronic diseases (adj. OR 3.92, AUC = 0.712). At 1-year follow-up, the overall incidence of ADL disability increased with the impairment in IC domains higher than the increase in multimorbidity status. A higher impairment in IC domains showed higher odds of incidence ADL disability for impairment in 2 or ≥3 IC domains (adj. OR 2.32 for impairment in ≥3 domains, adj. OR 1.43 for impairment in two domains, AUC = 0.685). Only subjects who had ≥3 chronic diseases had higher odds of 1-year incident ADL disability (adj. OR 1.73, AUC = 0.681) that was statistically significant.

**Conclusion:** Our results imply that a function-centered construct could have higher predictability of disability compared to the multimorbidity status in community older people. Our results need to be confirmed by studies with longer follow-up.

## Introduction

The disability-free life expectancy has not increased at the same pace as the life expectancy in humans (1). There is an increasing notion in geriatrics that the traditional disease-centered approach may be inadequate to meet the healthcare needs of older adults (2, 3). Strategies that promote “healthy aging” could assist in reducing the burden of disability and dependency in old age. The WHO defines healthy aging as the process of maintaining functional ability that enables well-being in old age (1, 4). Healthy aging is determined by intrinsic capacity (IC) and the environment (i.e., extrinsic factors) of an individual. The WHO introduced the concept of IC through its ambitious and innovative care plan known as the Integrated Care for Older Person (ICOPE) (4), which has a great potential to improve geriatric care even in settings without adequate geriatric medicine expertise. IC is defined as the composite of all physical and mental capacities of an individual. In other words, maintaining IC throughout life may serve as a meaningful approach to avoid dependency in old age by achieving optimal functional ability. Early detection and prevention of disability or dependency may be needed to maintain autonomy in old age.

Older adults with one or more chronic diseases or having multimorbidity are known to be at increased risk of disability (5). There is a high prevalence of multimorbidity in community-dwelling older adults (5, 6). A complex and persisting interplay between the aging process and disease is known to exist; hence, approaches based on the mere treatment of diseases may be inadequate to avoid the disability cascade. Strategies, such as enhancing or maintaining IC throughout life, could play an important role in improving the lives of older adults. However, research on IC is limited. There is very little evidence to confirm that this novel construct could serve its purpose as signified by the WHO ICOPE approach. Prior studies have shown IC to be able to predict poor health outcomes in nursing home residents (7) and to predict loss of functions in the English Longitudinal Study of Aging (ELSA) cohort (8). Two cross-sectional studies in China have shown IC to be associated with various adverse events in older adults (9, 10). Another study also attempted to validate the IC construct in a Chinese population, but the study population was from a single community (11). Moreover, it remains yet to be confirmed if this function-centered construct could be better than the traditional disease-centered approach in determining future disability in a representative community-dwelling older population, particularly in the Chinese population, which bears the largest aging population of the world.

We aimed to estimate the status of IC and examine whether impairment in IC predicts subsequent 1-year disability in a representative community-dwelling Chinese older population. We hypothesized that a function-centered construct, such as IC, could predict disability better than a disease-based approach, i.e., multimorbidity status.

## Methods

### Study Participants

**Figure 1:**
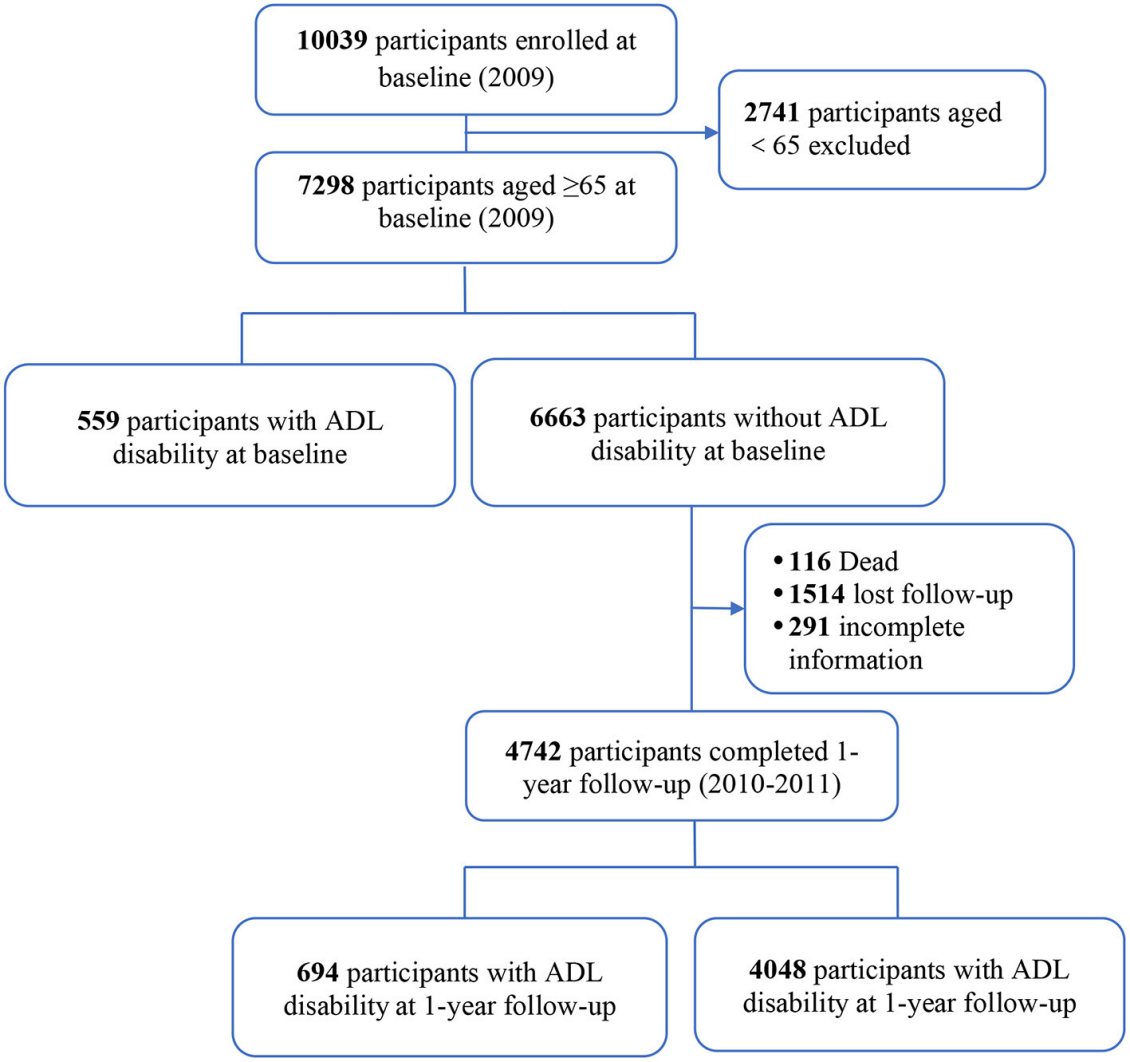
Flowchart of the study population.

This study participants were from the Beijing Longitudinal Study on Aging II (BLSA II), a representative community-dwelling older population cohort. The details on study design and cohort profile have been previously described (12, 13). In brief, 10,039 adults aged 55 years and older were selected using a multistage-randomized cluster sampling method from three urban districts and one rural county in the Beijing region. Participants were interviewed face to face by trained clinicians. For this current analysis, 7,298 subjects aged 65 years and older were included (Figure 1). The research and ethics committee of Xuanwu Hospital of Capital Medical University approved this research, and each participant provided written informed consent.

## Measures

### Intrinsic Capacity

According to the WHO ICOPE guideline (14), IC included five domains: locomotion, vitality, sensory (hearing and vision), cognition, and psychological capacity. We selected commonly used and well-validated scales for each domain, and all the scores were dichotomized as 1 = “impaired” and 0 = “not impaired.”

#### Locomotion

Locomotion was evaluated by the Tinetti score (15), which is also generally used to assess mobility, balance, gait, and predict falls in older people. The Tinetti score consists of 13 maneuvers and the score ranges from 0 to 26 (higher is better). The Tinetti test score <24 was considered as an impairment in locomotor capacity.

#### Vitality

Vitality was assessed using the Mini-Nutritional Assessment (MNA) scale (16), MNA is composed of 18 items with a maximum score of 30 (higher is better). MNA score <24 was considered as an impairment in vitality.

#### Sensory

The sensory capacity domain included vision and hearing impairments. Participants were asked if they experienced any recent decline in vision and hearing. A positive answer to a recent decline in vision or hearing impairments was considered as an impairment in sensory capacity. Self-reported hearing loss has been suggested to be useful where audiometry is not available (17), and self-reported vision impairment has been used as a measure of visual loss in prior IC studies (9).

#### Cognition

Cognition was evaluated using the Mini-Mental State Examination (MMSE) (18). MMSE test is composed of 11 items with a maximum score of 30 (higher is better). MMSE score <24 was considered as having an impairment in cognition.

#### Psychology

The psychological domain was evaluated using the 15-item Geriatric Depression Scale (GDS-15), which identifies depressive symptoms in older people with scores varying from 0 to 15 (higher is worse) (19). GDS-15 score ≥8 was considered as having a psychological impairment.

### Multimorbidity Status

Self-reported history of chronic diseases was collected using a single question “Have you been ever diagnosed with any of the following diseases by a doctor?” For our current study, we included six chronic diseases that were most common among the study population including hypertension, diabetes mellitus, cardiovascular disease (CVD), stroke, tumor, and chronic obstructive pulmonary (COPD). The total number of chronic diseases was categorized into four groups: 0, 1, 2, and ≥3 in our analysis.

### Other Covariates

Other covariates included three age groups (65–74, 75–84, and ≥85 years), sex (female and male), education (middle school or below vs. higher education), and marital status (currently married vs. others).

### Outcome Variable

#### Disability

Disability was assessed using the Barthel Index for basic activities of daily living (ADL) (20). Barthel index included 10 daily living tasks (feeding, bathing, grooming, dressing, bowels, bladder, toilet use, transfers, mobility, and climbing stairs). Subjects who had limitations in at least one task were considered as having ADL disability.

### Statistical Analyses

For continuous variables, arithmetic means *t*-tests were used to compare between groups. For categorical variables χ^2^ test was used to compare the groups. The prevalence of IC impairment was estimated by the proportion of subjects who had an impairment in at least one domain of IC at baseline. The chi-square tests were used to describe the associations between demographic characters and other subgroups with categories of IC impairment. We used logistic regressions to estimate the odds of ADL disability at baseline and 1-year incident ADL disability. Comparisons were made according to IC impairment and multimorbidity. Adjustments were made for age, sex, educational level, and marital status in the regression models. Education and marital status were included to consider the interaction between the environment and IC. Furthermore, these factors are also directly associated with the development and maintenance of IC. To assess the logistic model discrimination, c-statistics for the area under the curve (AUC) were calculated. The Hosmer–Lemeshow goodness-of-fit statistic was used to assess model calibration.

All analyses were performed using SAS version 9.3 (SAS Institute Inc., Cary, NC, USA) and all *p*-values are two-tailed. A *p*-value of <0.05 was considered as being statistically significant.

## Results

### Study Population

Of the 7,298 subjects aged 65 years and over enrolled in this study (Figure 1), we followed 6,663 participants without ADL disability for 1 year. At the 1-year follow-up visit, 4,742 (71.2%) subjects completed the study and 116 (1.7%) had died, and 1,514 (22.7%) lost to follow-up and 291 (4.4%) had incomplete data. Subjects who lost to follow-up had similar characteristics as those who were followed at 1 year (Supplementary Material 1).

### Baseline Characteristics

The characteristics of the study participants at baseline are presented in Table 1. At baseline, the mean age of the included 7,298 participants was 74.2 (±5.5) years, 60.9% were female, 45.9% had middle school or lower education, and 76.5% were currently married. About 35.9% had one of the six chronic diseases, 25% had two chronic diseases, and 10.9% had three or more chronic diseases. The proportion of IC impairment according to its individual domains was 11.1% in locomotion, 34.7% in vitality, 32.8% in sensory, 18.4% in cognition, and 11.8% in psychology.

**Table 1:** Characteristics of the study participants at baseline.

**Characteristics**	**Baseline** **(*n* = 7,298)**	**One-year follow-up** **(*n* = 4,742)**
	***N*** **(%)**	***N*** **(%)**
Mean age (SD)	74.2 (± 5.5)	
**Age group**		
65–74 y	4,266 (58.5)	2,940 (62.0)
75–84 y	2,785 (38.1)	1,692 (35.7)
≥85 y	247 (3.4)	110 (2.3)
**Sex**		
Female	4,447 (60.9)	2,849 (60.1)
Male	2,851 (39.1)	1,893 (39.9)
**Education**		
Middle school or below	3,348 (45.9)	2,079 (56.1)
High school or above	3,944 (54.1)	2,660 (43.9)
**Married**		
Yes	5,586 (76.5)	3,741 (78.9)
No	1,712 (23.5)	1,001 (21.1)
**Comorbidity**		
≥2 Chronic diseases	2,620 (35.9)	1,656 (35.0)
<2 Chronic diseases	4,653 (63.8)	3,073 (65.0)
**Number of chronic diseases**		
0	2,031 (27.8)	1,318 (27.9)
1	2,622 (35.9)	1,755 (37.1)
2	1,822 (25.0)	1,182 (25.0)
≥3	798 (10.9)	474 (10.0)
**Impairment in intrinsic capacity (IC) domains**		
Locomotion	807 (11.1)	342 (7.2)
Vitality	2,533 (34.7)	1,501 (31.7)
Sensory	2,390 (32.8)	1,483 (31.3)
Cognition	1,338 (18.4)	709 (15.0)
Psychology	806 (11.8)	454 (10.3)
**Activities of daily living (ADL) disability**		
Yes	559 (7.7)	603 (12.7)
No	6,663 (91.2)	4,139 (87.3)

### Prevalence of IC Impairment at Baseline With Risk Factors

Table 2 shows the global prevalence (i.e., impairment in at least one domain) of IC impairment and as categorized by the number of impairments in IC domains at baseline. Of the 7,298 participants, 4,709 (64.5%) had an impairment in at least one domain of IC. Among them, 34.5% had an impairment in only one domain, 19.9% in two domains, and 10.1% in three or more IC domains. The prevalence of the IC impairment increased with age (≥85 years had the highest decline of 80%) was higher in females, in individuals with low education, and who were currently unmarried. Individuals with any of the chronic diseases also showed impairments in IC.

**Table 2:** Prevalence of overall IC impairment and as categorized by the impairment in intrinsic capacity (IC) domains at baseline.

**Characteristics**	**IC impairment, *N* (%)**	***p***	**Number of impairment in IC domains**				***p***
			**0, *N* (%)**	**1, *N* (%)**	**2, *N* (%)**	**≥3, *N* (%)**	
Overall	4,709 (64.5)	–	2,589 (35.5)	2,519 (34.5)	1,454 (19.9)	736 (10.1)	–
**Age group**							
65–74 y	2,589 (58.7)	<0.001	1,762 (41.3)	1,497 (35.1)	743 (17.4)	264 (6.2)	<0.001
75–84 y	2,006 (72.0)		779 (28.0)	943 (33.9)	665 (23.9)	398 (14.2)	
≥85 y	199 (80.6)		48 (19.4)	79 (32.0)	46 (18.6)	74 (30.0)	
**Sex**							
Female	2,996 (67.4)	<0.001	1,451 (32.6)	1,488 (33.5)	991 (22.3)	517 (11.6)	<0.001
Male	1,713 (60.1)		1,138 (39.9)	1,031 (36.2)	463 (16.2)	219 (7.7)	
**Education**							
≤ Middle school	2,449 (73.1)	<0.001	899 (26.9)	1,142 (34.1)	837 (25.0)	470 (14.0)	<0.001
>Middle school	2,257 (57.2)		1,687 (42.8)	1,375 (34.9)	616 (15.6)	266 (6.7)	
**Married**							
Yes	3,449 (61.7)	<0.001	2,137 (38.3)	1,937 (34.7)	1,047 (18.7)	465 (8.3)	<0.001
No	1,260 (73.6)		452 (26.4)	582 (34.0)	407 (23.8)	271 (15.8)	
no	4,641 (64.4)		2,564 (35.6)	2,487 (34.5)	1,434 (19.9)	720 (10.0)	
**Comorbidity**							
≥2 Chronic diseases	1,876 (71.6)	<0.001	744 (28.4)	891 (34.0)	610 (23.3)	375 (14.3)	<0.001
<2 Chronic diseases	2,817 (60.5)		1,836 (39.5)	1,618 (37.8)	839 (18.0)	360 (7.7)	
**Number of chronic diseases**							
0	1,159 (57.1)	<0.001	872 (42.9)	670 (33.0)	347 (17.1)	142 (7.0)	<0.001
1	1,658 (63.2)		964 (36.8)	948 (36.2)	492 (18.8)	218 (8.3)	
2	1,291 (70.9)		531 (29.1)	647 (35.5)	406 (22.3)	238 (13.1)	
≥3	585 (73.3)		213 (26.7)	244 (30.6)	204 (25.6)	137 (17.1)	

### Prevalence of ADL Disability at Baseline

**Figure 2:**
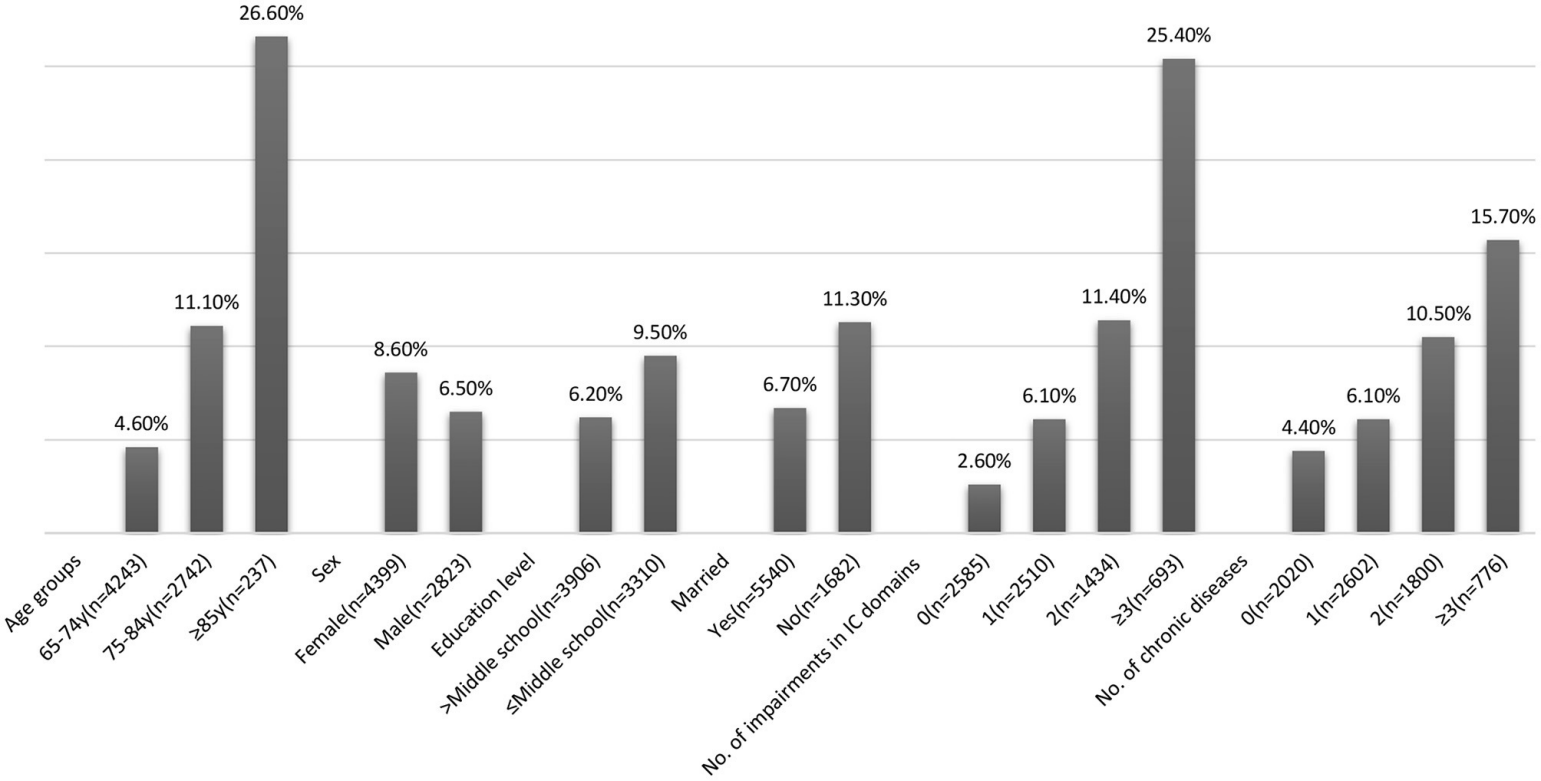
Prevalence of activities of daily living (ADL) disability.

The overall prevalence of ADL disability at baseline was 7.7% and increased with age, higher in females, in individuals with low education, and those who were currently unmarried (Figure 2). The ADL disability increased with the number of chronic diseases (15.7% in ≥3 chronic diseases vs. 6.1% with one chronic disease). Similarly, individuals with IC impairment had higher ADL disability (10.6 vs. 2.6% with no IC impairment). Disability increased with impairments in multiple domains of IC (30.4% in ≥3 domains vs. 6.1% with impairment in one domain). Locomotion was the domain with the highest rate of ADL disability (30.9%), and vitality was the domain with the lowest rate of ADL disability (11.6%).

### Association of IC Impairment and Multimorbidity Status With ADL Disability at Baseline

Subjects with a higher number of chronic diseases had higher odds of ADL disability [adj. odds ratio (OR) 3.92, 95%CI = 2.92–5.27 for ≥3 chronic diseases vs. adj. OR 1.38, 95%CI = 1.06–1.82 for one chronic disease]. The AUC for the unadjusted model of multimorbidity was 0.63 and 0.712 for the adjusted model (Table 3).

**Table 3:** Logistic regression to determine the odds of activities of daily living (ADL) disability at baseline.

	**ORs for the prevalence of ADL disability (95% CI)**							
	**Unadjusted**	***p***	**Model 1**	***p***	**Model 2**	***p***	**Model 3**	***p***
**Age group**								
65–74 y	Ref		Ref		Ref		Ref	
75–84 y	2.61 (2.16, 3.15)	0.586	2.02 (1.66, 2.47)	0.460	2.30 (1.89, 2.80)	0.135	1.95 (1.60, 3.23)	0.165
≥85 y	7.60 (5.48, 10.45)	<0.001	4.79 (3.34, 6.81)	<0.001	7.23 (5.10, 10.16)	<0.001	5.13 (3.57, 7.33)	<0.001
**Sex**								
Male	Ref		Ref		Ref		Ref	
Female	1.35 (1.12, 1.62)	0.001	1.22 (0.99, 1.49)	<0.062	1.33 (1.09, 1.63)	<0.006	1.22 (0.99, 1.50)	0.061
**Education**								
High school or above	Ref		Ref		Ref		Ref	
Middle school or below	1.59 (1.33, 1.89)	<0.001	0.986 (0.82, 1.20)	0.885	1.28 (1.06, 1.54)	0.010	1.04 (0.85, 1.26)	0.722
**Married**								
Yes	Ref		Ref		Ref		Ref	
No	1.79 (1.48, 2.14)	<0.001	1.14 (0.93, 1.40)	0.203	1.24 (1.01, 1.52)	0.037	1.14 (0.93, 1.40)	0.209
**No. of chronic diseases** ***^a^***								
0	Ref		/		Ref		/	
1	1.43 (1.10, 1.87)	<0.001	/		1.38 (1.06, 1.82)	<0.001	1.34 (1.02, 1.77)	0.001
2	2.58 (1.99, 3.36)	<0.001	/		2.36 (1.81, 3.10)	0.002	2.07 (1.58, 2.72)	0.014
≥3	4.10 (3.08, 5.48)	<0.001	/		3.92 (2.92, 5.27)	<0.001	3.16 (2.33, 4.29)	<0.001
**No. of impairments in IC domains** ***^b^***								
0	Ref		Ref		/		Ref	
1	2.48 (1.86, 3.34)	<0.001	2.26 (1.69, 3.06)	<0.001	/		2.17 (1.62, 2.94)	<0.001
2	4.93 (3.69, 6.65)	<0.001	4.23 (3.15, 5.74)	<0.001	/		3.84 (2.86, 5.22)	<0.001
≥3	12.99 (9.69, 17.61)	<0.001	9.51 (7.01, 13.02)	<0.001	/		8.24 (6.07, 11.31)	<0.001
**AUC**	0.630^a^/0.719^b^		0.751		0.712		0.767	

*ADL, activities of daily living; OR, odds ratio; No., number; IC, intrinsic capacity; AUC, area under the curve*.

a *Multimorbidity status: Chronic diseases include: hypertension, coronary artery disease, diabetes mellitus, stroke, tumor, and chronic obstructive pulmonary*.

b *Intrinsic capacity status: IC includes five domains: locomotion, vitality, sensory, cognition, and psychological*.

*Model 1: Adjustment for age, sex, education, and marriage to determine the odds of disability based on IC impairment*.

*Model 2: Adjustment for age, sex, education, and marriage to determine the odds of disability based on multimorbidity status*.

*Model 3: Adjustment for multimorbidity status, age, sex, education, and marriage to determine the odds of disability based on IC impairment*.

Impairments in multiple domains of IC showed higher odds of ADL disability (adj. OR 9.51, 95%CI = 7.01–13.02 for impairments in ≥3 domains vs. adj. OR 2.26, 95%CI = 1.69–3.06 for impairment in one domain). The association remained equally significant even after including adjustment for chronic diseases. The AUC for the unadjusted model of IC was 0.719 and 0.767 for the fully adjusted model.

### Incident ADL Disability at 1-year Follow-Up

**Figure 3:**
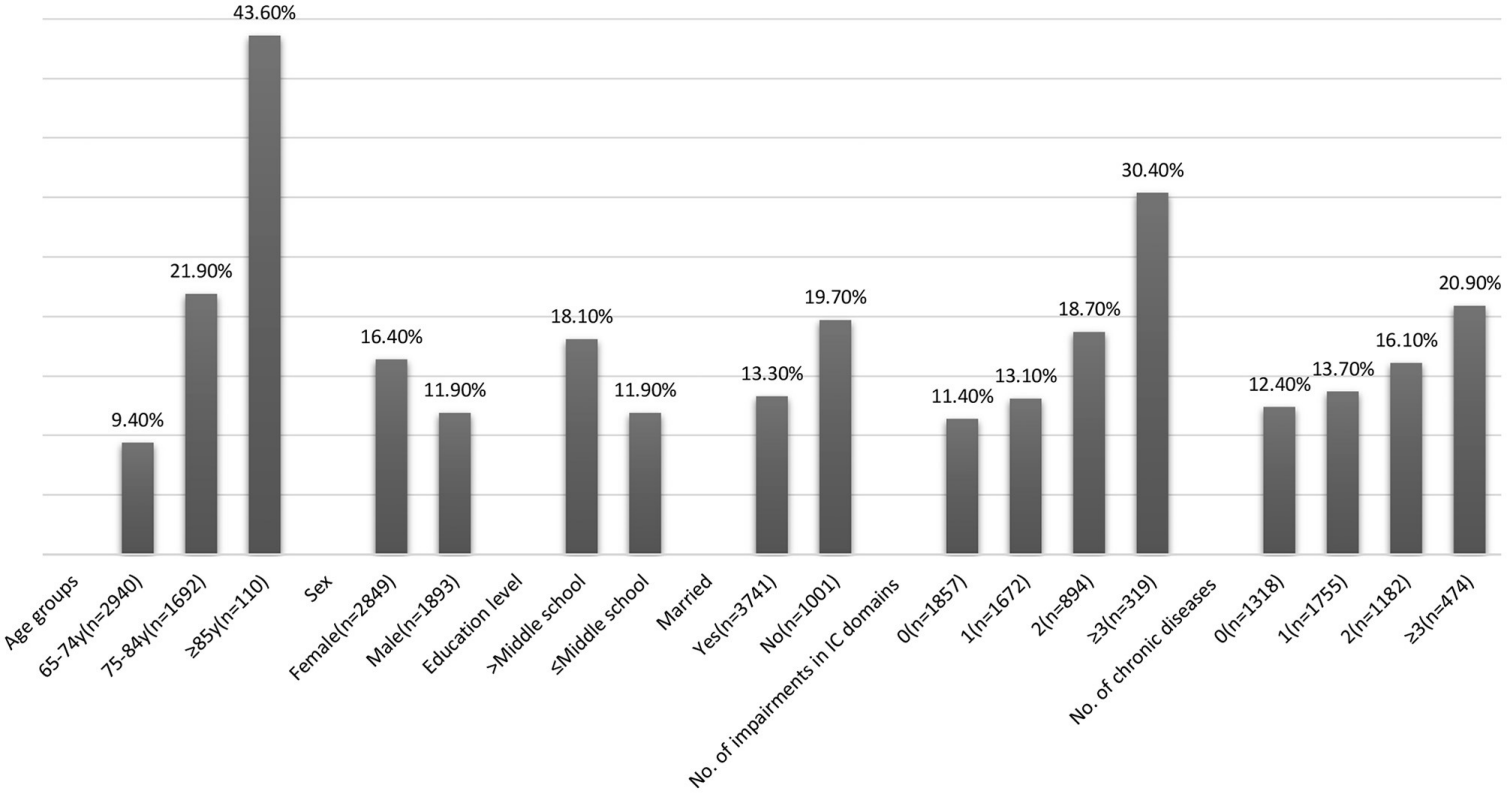
Incidence of activities of daily living (ADL) disability at 1-year follow-up.

At 1-year follow-up, 694 (14.6%) new onset of ADL disability was detected. The incidence of ADL disability increased with age was higher in females, in individuals with lower education, and those who were currently unmarried (Figure 3). The overall incidence of ADL disability showed an increasing trend with impairments in multiple domains of IC (30.4% in ≥3 domains vs. 13.7% with one domain) and the number of chronic diseases (20.9% in ≥3 chronic diseases vs. 12.4% with one chronic disease).

### Association of IC Impairment and Multimorbidity Status With 1-year Incident ADL Disability

Only subjects who had ≥3 chronic diseases had a significant odds of 1-year incident ADL disability (adj. OR 1.73, 95%CI = 1.30–2.30). Impairments in multiple domains of IC showed higher odds of incidence ADL disability; however, significance was observed only for impairments in 2 or ≥3 domains (adj. OR 2.32, 95%CI = 1.72–3.11 for impairments in ≥3 domains, adj. OR 1.43, 95%CI = 1.14–1.80 for impairments in two domains). The association remained equally significant even after including adjustment for chronic diseases. The AUC for the fully adjusted model of IC impairment was 0.691 and 0.681 for the model of multimorbidity status (Table 4).

**Table 4:** Logistic regression to determine the odds of activities of daily living (ADL) disability at 1-year follow-up.

	**ORs for the incidence of ADL disability (95% CI)**							
	**Unadjusted**	***p***	**Model 1**	***p***	**Model 2**	***p***	**Model 3**	***p***
**Age group**								
65–74 y	Ref		Ref		Ref		Ref	
75–84 y	2.72 (2.30, 3.23)	0.956	2.58 (2.16, 3.08)	0.870	2.64 (2.22, 3.16)	0.631	2.52 (2.11, 3.02)	0.678
≥85 y	7.50 (5.03, 11.14)	<0.001	6.92 (4.56, 10.45)	<0.001	7.84 (5.18, 11.79)	<0.001	7.05 (4.64, 10.65)	<0.001
**Sex**								
Male	Ref		Ref		Ref		Ref	
Female	1.45 (1.22, 1.72)	<0.001	1.49 (1.24, 1.80)	<0.001	1.54 (1.28, 1.86)	<0.001	1.49 (1.23, 1.80)	<0.001
**Education**								
High school or above	Ref		Ref		Ref		Ref	
Middle school or below	1.63 (1.39, 1.92)	<0.001	1.17 (0.98, 1.39)	0.091	1.14 (0.94, 1.39)	0.009	1.18 (0.99, 1.41)	0.066
**Married**								
Yes	Ref		Ref		Ref		Ref	
No	1.60 (1.33, 1.92)	<0.001	1.09 (0.89, 1.33)	0.391	1.26 (1.06, 1.50)	0.182	1.10 (0.90, 1.34)	0.337
**No. of chronic diseases** ***^a^***								
0	Ref		/		Ref		/	
1	1.12 (0.91, 1.39)	0.028	/		1.08 (0.87, 1.35)	0.063	1.07 (0.86, 1.33)	0.097
2	1.35 (1.71, 1.69)	<0.001	/		1.21 (0.96, 1.53)	0.086	1.17 (0.93, 1.48)	0.794
≥3	1.86 (1.41, 2.44)	<0.001	/		1.73 (1.30, 2.30)	<0.001	1.64 (1.23, 2.18)	0.001
**No. of impairments in IC domains** ***^b^***								
0	Ref		Ref		/		Ref	
1	1.18 (0.96, 1.44)	0.002	1.07 (0.88, 1.32)	0.014	/		1.07 (0.87, 1.31)	0.0195
2	1.79 (1.44, 2.24)	<0.001	1.43 (1.14, 1.80)	<0.001	/		1.40 (1.11, 1.76)	<0.001
≥3	3.41 (2.58, 4.50)	<0.001	2.32 (1.72, 3.11)	<0.001	/		2.23 (1.66, 2.99)	<0.001
**AUC**	0.549^a^/0.589^b^		0.685		0.681		0.691	

*ADL, activities of daily living; OR, odds ratio; No., number; ref, reference; IC, intrinsic capacity; AUC, area under the curve*.

a *Multimorbidity status: Chronic diseases include: hypertension, coronary artery disease, diabetes mellitus, stroke, tumor, and chronic obstructive pulmonary*.

b *Intrinsic capacity status: IC includes five domains: locomotion, vitality, sensory, cognition, and psychological*.

*Model 1: Adjustment for age, sex, education, and marriage to determine the odds of disability based on IC impairment*.

*Model 2: Adjustment for age, sex, education, and marriage to determine the odds of disability based on multimorbidity status*.

*Model 3: Adjustment for multimorbidity status, age, sex, education, and marriage to determine the odds of disability based on IC impairment*.

## Discussion

In this study, we investigated the association of IC impairment and multimorbidity with disability using a representative sample of community-dwelling older adults in China. Our findings showed IC impairment to be associated with higher odds of disability both cross-sectionally and at 1-year follow-up compared to multimorbidity. Odds of disability increased with impairment in multiple domains of IC compared to the increase in the number of diseases. These findings support our hypothesis that a function-centered approach could provide better prognostic information on the process of disability beyond that contributed by the presence or absence of multiple chronic diseases in older adults.

Our study showed the global prevalence of IC impairment to be 64.5%, which is in agreement with a previous study (in Chinese) of relatively healthy inpatient population (10), and slightly higher than another study from a longitudinal cohort (9). IC impairment was the highest in the vitality domain (34.7%), followed by sensory (32.8%), cognition (18.4%), psychological (11.8%), and locomotion (11.1%). However, impairment according to individual domains of IC differed in the past studies. One of the reasons for such difference is the variations in the methods used for assessing individual domains of IC. Although the WHO ICOPE care plan has suggested certain screening methods for each domain of IC, the overall concept of IC remains to be validated in multiple populations (21). Hence, many previous studies and including our current study have used tools that are different from what the WHO has recommended but which equally capture the spectrum of each domain. For instance, vitality is a measure of physiologic factors (such as energy balance and metabolism) contributing to the IC of an individual. We have used MNA to assess vitality, whereas several techniques such as gait speed and grip strength may be equally effective in assessing vitality. It is undeniable that there is a necessity for robust and uniform approaches to measure IC (21), in particular, if we were to expect implementing IC in clinical settings soon (i.e., avoiding proliferation leading to ambiguities).

The WHO healthy aging framework divides the decline in IC into three periods: a period of relatively high and stable function and capacity, a period of declining capacity, and a period of significant loss in capacity and function indicated by dependency and disability (14). Our study supports this concept showing older individuals who have impairments in multiple domains of IC to have a higher prevalence of ADL disability (representing a state of significant loss in capacity). Indeed, longitudinal data with multiple follow-ups are needed to fully explore the trajectory of IC. The prevalence of the disability was also higher in those with impairments in multiple domains of IC compared to those with a higher number of diseases or multimorbidity. Our study also showed that subjects with the IC impairment had a higher odds of being disabled. Subjects who had impairments in three or more domains had almost 10 times higher risk of being disabled compared to those without any impairments in IC domains. However, the correlation between comorbidity status and ADL disability was not as stronger. Subjects with three or more diseases had about four times higher odds of being disabled. A similar trend was observed even while considering a 1-year incident disability. However, higher odds of incident ADL disability were associated with the presence of three or more chronic diseases and impairments in over two domains of IC. It should also be noted that although the AUCs for both models (i.e., IC and multimorbidity) were almost similar demonstrating uniform performance of the models. However, higher odds ratios in the model with IC impairment showed better predictability of ADL disability compared to the model with multimorbidity. These findings are in accordance with our hypothesis.

The relation between multimorbidity, IC, and disability was also highlighted in a previous study conducted in the ELSA cohort (8). The authors demonstrated that although multimorbidity too predicted incident disability, IC was far more superior, many of the personal characteristics contributing to the loss of function was mediated through IC including multimorbidity. Such findings could be explained through a recent theory that IC could be influenced not only by the environment but also through the level of physiologic reserve of an individual (22). Individuals with lower physiologic reserve or with impairments in IC (22) could experience poor recovery once exposed to stressors and as a result of the continuum of the aging process and diseases and may be vulnerable to being disabled, which is also in line with our results. Some studies on the subject have also shown IC impairment to be associated with disability including in the community-dwelling older population (9, 11) and hospitalized patients (7, 10).

Population aging has led to the emergence of geriatric medicine, particularly in countries such as China (23). Geriatricians have begun to advocate that now is the time to put an end to the disease-based approach (3) and initiate implementation of a function-based approach such as frailty to improve the care needs of older adults (2). Frailty, which is a geriatric syndrome characterized by reduced homeostasis and increased vulnerability to stressors (24), undeniably has stressed the need to focus on functions rather than treating a single disease. For example, China, which has the highest number of older people worldwide, has already enough studies on frailty (12, 25) to justify the need to prevent disability and maintain autonomy in old age (26). However, population aging is a positive aspect of human progress and instead of focusing on health deficits (or negative health attributes) such as the lauded concept of frailty, greater consideration is being given to the concepts that capture positive health attributes and empower older adults such as IC. Our findings have proven a function-centered approach (driven by positive health attributes) such as IC can effectively predict disability in older people better than disease-based approaches such as multimorbidity. The construct of IC holds a great potential to transform geriatric care worldwide including in regions without well-established geriatric medicine.

Our study has several limitations. Some of the recommended assessment methods were not available in our study cohort; hence, we used alternative methods to measure the IC domains. Nevertheless, the methods we used should equally capture the magnitude of all of these domains. Some of the subjects who lost to follow-up could be the ones who were already dependent; hence, the incidence rate of disability may have been underestimated. We used six chronic diseases that were most common in our study population to assess the severity of multimorbidity. However, different chronic diseases might not have the same weight while considering the effect of multimorbidity on disability (e.g., cardiovascular conditions vs. tumors and others or combined), hence could have influenced our findings. Furthermore, self-reported history of disease is also another limitation of our study, which might have impacted the multimorbidity status of the study population. Nevertheless, there are several strengths of this study. Our study was performed in a representative sample of the community-dwelling older population in the Beijing region. This sample included both urban and rural populations hence could better represent the Chinese aging scenario sample. To our knowledge, this is the first study to specifically compare IC (according to impairments in multiple domains) and multimorbidity (stratified by the number of diseases) in predicting future disability.

In conclusion, our findings imply that a function-centered construct could be more useful in predicting future disability beyond the traditional disease-based approach. Nevertheless, our findings need to be confirmed in future studies with much longer follow-ups. The WHO ICOPE care plan for older people is centered around the construct of IC. This care plan is proposed to help older people in achieving healthy aging, i.e., enable them to be independent and perform tasks that they value the most. The ICOPE approach highlights the need to reform geriatric care from a disease-centered approach to a function-centered approach, which has been justified from our study. This reform should commence from the very base level of healthcare, and priority should be given to the evaluation of IC instead of just treating disease in primary care while examining an old patient. Moreover, public health strategies to maintain IC individuals throughout life course should be developed that are easy to implement and are cost-effective.

## Data Availability Statement

The datasets generated from this study may be available upon reasonable request to the corresponding author.

## Ethics Statement

The studies involving human participants were reviewed and approved by Ethics Committee of Xuanwu Hospital of Capital Medical University. The patients/participants provided their written informed consent to participate in this study.

## Author Contributions

JZ, JC, PC, and LM responsible for study design. JZ and JC responsible for manuscript preparation. JZ, ZZ, JC, YC, and LM responsible for acquisition of subjects and data, and analysis and interpretation of data. JC and PC critically reviewed the article. All authors approved the final version of the manuscript.

## Funding

This study was supported by The National Key R&D Program of China, No. 2018YFC1312001, 2017YFC0840105, and 2017ZX09304018; and the Key Realm R&D Program of Guangdong Province 2018B030337001.

## Conflict of Interest

The authors declare that the research was conducted in the absence of any commercial or financial relationships that could be construed as a potential conflict of interest.

## Publisher's Note

All claims expressed in this article are solely those of the authors and do not necessarily represent those of their affiliated organizations, or those of the publisher, the editors and the reviewers. Any product that may be evaluated in this article, or claim that may be made by its manufacturer, is not guaranteed or endorsed by the publisher.
