# Supplementary material for: Intrinsic Capacity vs. Multimorbidity: A Function-Centered Construct Predicts Disability Better Than a Disease-Based Approach in a Community-Dwelling Older Population Cohort
Source: Front Med (Lausanne). 2021 Sep 28;8:753295. doi: 10.3389/fmed.2021.753295 (PMC8505775; doi:10.3389/fmed.2021.753295)
Supplement: Supplementary file 1 [file Table_1.DOCX]

| **Characteristics** | **Baseline**  **(n=7298)** | **Baseline**  **(n=6663)** | **One-year follow-up (n=4742)** | **Lost to**  **follow-up (n=1921)** |
| --- | --- | --- | --- | --- |
|  | **N (%)** | **N (%)** | **N (%)** | **N (%)** |
| **Mean age (SD)** | 74.2(5.5) |  |  |  |
| **Age group** |  |  |  |  |
| 65-74y | 4266(58.5) | 4050(60.8) | 2940(62.0) | 1110(57.8) |
| 75-84y | 2785(38.1) | 2439(36.6) | 1692(35.7) | 747(38.9) |
| ≥85y | 247(3.4) | 174(2.6) | 110(2.3) | 64(3.3) |
| **Sex** |  |  |  |  |
| Female | 4447(60.9) | 4023(60.4) | 2849(60.1) | 1174(61.1) |
| Male | 2851(39.1) | 2640(39.6) | 1893(39.9) | 747(38.9) |
| **Education** |  |  |  |  |
| Middle school or below | 3348(45.9) | 2995(44.9) | 2079(56.1) | 916(47.7) |
| High school or above | 3944(54.1) | 3663(55.0) | 2660(43.9) | 1003(52.2) |
| **Married** |  |  |  |  |
| Yes | 5586(76.5) | 5171(77.6) | 3741(78.9) | 1430(74.4) |
| No | 1712(23.5) | 1492(22.4) | 1001(21.1) | 491(25.6) |
| **No. of chronic diseases** |  |  |  |  |
| 0 | 2031(27.8) | 1932(29.0) | 1318(27.9) | 614(32.0) |
| 1 | 2622(35.9) | 2443(36.7) | 1755(37.1) | 688(35.8) |
| 2 | 1822(25.0) | 1611(24.2) | 1182(25.0) | 429(22.3) |
| ≥3 | 798(10.9) | 654(9.8) | 474(10.0) | 180(9.4) |
| **Chronic diseases** |  |  |  |  |
| HT | 4243(58.3) | 3818(57.3) | 2784(58.9) | 1034(53.8) |
| CAD | 1794(24.7) | 1578(23.7) | 1158(24.5) | 420 (21.9) |
| DM | 1629(22.4) | 1420(21.3) | 1016(21.5) | 404(21.0) |
| Stroke | 901(12.4) | 714(10.7) | 531(11.2) | 183(9.5) |
| Tumor | 152(2.1) | 131(2.0) | 94(2.0) | 37(1.9) |
| COPD | 68(0.9) | 53(0.8) | 29(0.6) | 24(1.3) |
| **Impairment in IC domains** |  |  |  |  |
| Locomotion | 807(11.1) | 529(7.9) | 342(7.2) | 187(9.7) |
| Vitality | 2533(34.7) | 2189(32.9) | 1501(31.7) | 688(35.8) |
| Sensory | 2390(32.8) | 2081(31.2) | 1483(31.3) | 598(31.1) |
| Cognition | 1338(18.4) | 1098(16.5) | 709(15.0) | 389(20.3) |
| Psychology | 806(11.8) | 668(10.0) | 454(10.3) | 214(11.1) |

**Supplementary file: Comparison of the characteristics of the population who lost to follow-up at one year with other population.**
